# Multi-Omics Identified THDCA as a Key Contributor to Hyperlipidemia and as a Potential Therapeutic Agent

**DOI:** 10.31083/j.rcm2409248

**Published:** 2023-09-05

**Authors:** Zhaohuan Lou, Liping Han, Yuanguo Qu, Aizhen Zhou, He Ye, Meiqiu Yan, Bin Cheng, Muyi Liu, Tao Jiang, Jianbo Huang

**Affiliations:** ^1^School of Pharmaceutical Sciences, Zhejiang Chinese Medical University, 310053 Hangzhou, Zhejiang, China; ^2^School of Basic Medical Sciences, Zhejiang Chinese Medical University, 310053 Hangzhou, Zhejiang, China; ^3^School of Chinese Materia Medica, Zhejiang Pharmaceutical University, 315000 Ningbo, Zhejiang, China; ^4^Pharmacy of Traditional Chinese Medicine, Zhejiang Hospital, 310013 Hangzhou, Zhejiang, China; ^5^Biological Sciences Department, Computer Science Department, Purdue University, West Lafayette, IN 47907, USA

**Keywords:** THDCA, hyperlipidemia, intestinal flora, bile acids

## Abstract

**Background:**

In recent years, with the change in human dietary habits, hyperlipidemia (HLP) has become a common chronic disease. Hyperlipidemia is closely related to the incidence of cardiovascular diseases. Due to the increasing incidence and mortality from cardiovascular diseases, it is imperative to develop new medications for reducing lipid levels. With the aim of discovering new treatment options for hyperlipidemia, we conducted a multi-omics analysis of a potential endogenous bile acid compound.

**Methods:**

Two hyperlipidemia models were established by feeding rats and mice with a high-fat diet. Serum and fecal specimens of rats with hyperlipidemia were collected. Through the combined analysis of lipid metabolism sequencing, 16S RNA intestinal flora sequencing, and bile acid targeted metabolism sequencing, taurohyodeoxycholic acid (THDCA) was found to be a potential lipid-lowering compound. A mouse hyperlipidemia model was developed to verify the anti-hyperlipidemia function of THDCA.

**Results:**

Analysis of serum lipid metabolites revealed that the synthesis of bile acid was one of the metabolic pathways that showed significant alterations. 16S RNA sequencing of intestinal flora also found that high-fat diet intake greatly influenced both primary and secondary bile acid biosynthesis. Analysis of bile acid metabolites in the serum and liver tissue found that THDCA in the secondary bile acids is a potential biomarker of hyperlipidemia. Verification experiments in mice confirmed the beneficial function of THDCA in lowering abnormal lipid levels induced by a high-fat diet.

**Conclusions:**

THDCA has been identified as a biomarker of hyperlipidemia and has shown potential for the treatment of hyperlipidemia.

## 1. Introduction

Due to improved standards of living and a growing aging population globally, there has been a significant rise in the incidence of metabolic disorders such as obesity, coronary heart disease, and myocardial infarction [1, 2, 3]. Elevated blood lipid levels are frequently found in these chronic metabolic diseases. Hyperlipidemia is characterized by altered levels of cholesterol components, including high levels of total cholesterol (TC), low-density lipoprotein cholesterol (LDL-C), and triglycerides (TG), as well as reduced levels of high-density lipoprotein cholesterol (HDL-C) [4]. The determinants of hyperlipidemia are derived from a complex interrelationship between genetic predisposition and environmental influences such as diet, lifestyle, and geographical location. The underlying mechanisms remain a subject of ongoing investigation [5]. The prevalence and mortality rates of hyperlipidemia-related diseases, such as coronary artery disease, are rapidly increasing, and account for approximately half of all global deaths [6]. Early detection and treatment have a positive effect on the prognosis of hyperlipidemia [7]. Currently, lipid-lowering drugs commonly used in clinical practice mainly include statins, niacin, and fibrates. However, all of them carry certain side effects, such as myositis, fatal kidney injury [8], gastrointestinal symptoms, headache, and insomnia [9]. Thus, there is a critical need to find alternative treatments for hyperlipidemia.

**Fig. 1.**
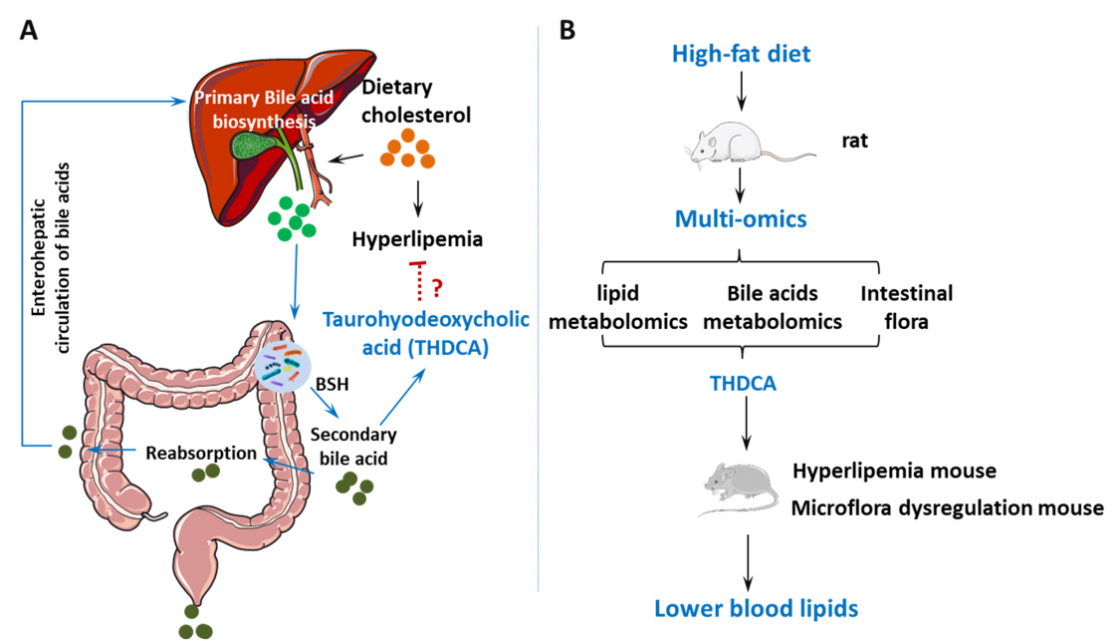
**Illustration of introduction (A) and experiment plan (B)**. BSH, bile salt hydrolase.

Studies have shown that the consumption of a high-fat diet (HFD) can alter bile acid (BA) metabolism and negatively impact the gut microbiota [10]. The abnormal interplay between the gut microbial community and BA metabolism is a critical factor that contributes to hyperlipidemia [11]. BAs play a crucial role in facilitating the emulsification and absorption of dietary lipids [12], and in maintaining serum cholesterol balance through the regulation of the enterohepatic circulation of BAs [13]. Different types of BAs act as signaling molecules on regulating lipid levels of the host via its receptor farnesoid X receptor (FXR) and the Takeda G protein-coupled bile acid receptor (TGR5) mediated signaling pathway [14], and are considered as a potential target in the treatment of hyperlipidemia [15]. However, the use of BAs as a biomarker for hyperlipidemia requires further investigation. Intestinal microflora are involved in the occurrence and development of hyperlipidemia by regulating the biosynthesis of secondary BAs, which then has an impact on BA enterohepatic circulation [16]. Studies have shown that hyperlipidemia is often accompanied by an imbalance between the contents of primary BAs (such as Lithocholic Acid (LCA)) and secondary BAs (such as taurohyodeoxycholic acid (THDCA)) [17, 18]. Changes in BAs may be a novel target for the treatment of hyperlipidemia [19]. In this study, we evaluated lipid metabolomics, 16S RNA analysis of intestinal flora, and BA metabolomics to determine potential targets that would lower lipid levels in a hyperlipidemia rat model induced with an HFD. These potential targets were then verified in a hyperlipidemia mouse model (Fig. 1).

## 2. Materials and Methods

### 2.1 Materials

#### 2.1.1 Animals

Sixteen male Sprague-Dawley (SD) rats, with body weight of 180–200 g (6 weeks old), were provided by the Animal Supply Center of Zhejiang Academy of Medical Science [Hangzhou, China; License number: SCXK(Zhe)2014-0001], and 60 male C57BL/6 mice with body weight of 18–21 g (6 weeks old) were purchased from Shanghai Slick Animal Laboratory Co., Ltd. [License number: SCXK(Shanghai)2017-0005]. All animals were housed in the Laboratory Animal Center of Zhejiang Chinese Medicine University. Animal procedures were carried out following the Animals (Scientific Procedures) Act 1986, and all experiments were approved by the Institutional Animal Care and Use Committee (ethics approval number: IACUC-20210726-07).

#### 2.1.2 Drugs and Reagents

Taurohyodeoxycholic acid sodium salt hydrate (item No.B16A10K94146, Shanghai yuanye Bio-Technology Co., Shanghai, China); Metronidazole (item No.B25D11R135700, Shanghai yuanye Bio-Technology Co., Shanghai, China); Neomycin sulfate (item No.20210708 Bellinger Biomedical Research Institute Co., Beijing, China); Vancomycin hydrochloride (item No.C12446387, Shanghai Macklin Biochemical Co., Shanghai, China); Ampicillin (item No.C12029421, Shanghai Macklin Biochemical Co., Shanghai, China); TC, TG, HDL and LDL kits (item No.210901201, 201106202, 210610201, 210128202, Ningbo Meikang Biotechnology Co., Ningbo, China); Total bile acid (TBA) test kit (item No.E003-2-1, Nanjing Jiancheng Biotechnology Co., Nanjing, China); SAKURA Frozen Section Embedding Agent (item No. 75930020191031, Japan SAKURA Co., Tokyo, Japan); Oil Red O staining kit (item No. G1261, Beijing Solarbio Science & Technology Co., Beijing, China); E.Z.N.A.® soil DNA Kit (Omega Bio-Tek Co., Norcross, GA, USA).

#### 2.1.3 Instruments

TBA-40FR automatic Biochemical analyzer (Toshiba Medical Systems Co., Otahara City, Tochigi Prefecture, Japan); Microc117 microcentrifuge (Thermo Fisher Scientific Inc Co., MA, USA); FJ-200 high speed dispersing homogenizer (Shanghai Specimen Mould Factory, Shanghai, China); SAKURA Tissue-Tek VIP 5Jr Automatic dehydrator (Japan SAKURA Co., Tokyo, Japan); MEIKO EC360 Embedding Machine (Meikang Equipment manufacturing Gaochun County Co., Nanjing, China.); LEI-CARM2245 slicer (Leica Biosystems, Nuslow, Germany); Biological Microscope (Guangzhou Mingmei Technology Co., Guangzhou, China); NX70 frozen slicer (Thermo Fisher Scientific Inc Co., MA, USA); Waters 2777C UPLC system (Waters Co., Milford, MA, USA); Mass spectrometer Xevo G2-XS QTOF (Waters, Milford, MA, USA).

### 2.2 Methods

#### 2.2.1 Treatment Groups

Sixteen male SD rats were divided equally into a normal control group (NC) and a hyperlipidemia (HLP) model group. An HLP model was established by feeding SD rats an HFD for four weeks, as per our previous experiment [11]. Following the experiment, all the rats were sacrificed, and serum lipid metabolite levels, intestinal flora 16S RNA sequencing, and BA metabolite analysis were carried out to screen the potential BA compound as a biomarker and therapy target of HLP. The verification of THDCA on lowering lipid levels of HLP was carried out in mice experiments. After seven days of adaptation, 60 male C57BL/6 mice were randomly divided into five groups, each with 12 mice: a normal group, HFD model group, THDCA group, microflora dysregulation group, and a THDCA + microflora dysregulation group. The normal group was fed a normal diet, and mice in the other groups were fed with an HFD to induce HLP. Microflora dysbiosis was induced by giving mice a combination of ampicillin (10 mg/kg/d), metronidazole (10 mg/kg/d), neomycin trisulfate (10 mg/kg/d), and vancomycin (5 mg/kg/d) continuously for two weeks. The normal group and the HFD model group were administered intragastric (i.g.) deionized water, and the other groups were given i.g. with THDCA 100 mg/kg/day.

#### 2.2.2 General Observations 

The activities of the mice were observed daily, and their body weights were measured every week. Following the experiment, the body fat content and liver weight of mice in each group were measured after the animals were sacrificed.

#### 2.2.3 Blood Lipid Levels

Blood samples were collected from the posterior orbital venous plexus of mice at the third and fifth weeks. After centrifuging at 4 °C (206 ×*g*) for 15 min, the serum was separated and stored at –80 °C. Serum TC, LDL-C, HDL-C, and TG were measured by a fully automatic blood biochemistry analyzer (TBA-40FR; Toshiba Medical Systems Corporation, Otawara, Japan) with commercial biochemical kits. Serum total bile acid (TBA) content was detected by a biochemical kit according to the manufacturer’s instructions.

#### 2.2.4 Histopathological Techniques 

H&E staining was performed to observe histopathological changes in the liver tissues. After conducting the experiment and euthanizing the mice, the right lobe of each mouse’s liver was harvested and dissected into smaller pieces. Liver tissues were fixed with 10% neutral formalin buffer solution for 48 h, then dehydrated with ethanol, embedded in paraffin, and sliced into 4 µm sections. The sections were dewaxed and stained with hematoxylin and eosin for histological evaluation.

#### 2.2.5 Oil Red O Staining

Oil red O staining was used to visualize the lipid accumulation in the livers of the mice. The left lobe of the liver was collected and cut into sections. Liver tissue was embedded with OTC embedding agent, and sections of approximately 8 µm in thickness were cut by a frozen slicer. The sections were washed in distilled water, soaked with 60% isopropyl alcohol for 2 min, stained with oil red O working solution for 2–5 min, washed with 60% isopropyl alcohol, re-stained with hematoxylin within 1min, followed by hydrochloric acid alcohol differentiation for 1–5 s, then sealed with glycerine gelatin. Lipid deposition in liver tissue was observed under an optical microscope.

#### 2.2.6 Serum Lipid Profile

Before Ultra Performance Liquid Chromatography-Quadrupole-Time of Flight-Mass Spectrometry (UPLC-QTOF-MS) analysis, metabolites in 40 µL rats’ serum sample were extracted by adding 120 µL of methanol and vortexing for 1 min. The mixture was maintained at –20 °C for 30 min and centrifuged at 4000 ×*g* for 20 min at 4 °C.The supernatant of each sample was transferred to a vial tube and diluted with 180 µL 50% (v/v) methanol, then filtered by a syringe filter before UPLC-QTOF-MS analysis. UPLC-QTOF-MS analysis was performed by a Waters 2777C UPLC system coupled to a Waters and Xevo G2-XS QTOF Mass Spectrometer. The QTOF operated in both positive (ESI+, electrospray ionization-positive ion mode) and negative (ESI–, electrospray ionization-negative ion mode) ion modes. For ESI+, the capillary and sampling cone voltages were set at 2 kV and 40 V in positive ion mode, and at 2 kV and 40 V in ESI–, respectively. The mass spectrometry data were acquired in Centroid MSE mode.

All data were presented as means ± SD. Peak extraction and identification were carried out by Progenesis QI (version 2.2; Nonlinear Dynamics, Quayside, Newcastle Upon Tyne, UK). Multivariate statistical analysis was performed using SIMCA-P 11.5 software (Umetrics, Umeå, Sweden). Principal component analysis (PCA) was used initially in all samples to observe the general separation. In-depth analysis of metabolite identification was based on the KEGG database (http://www.genome.jp/kegg/).

#### 2.2.7 BA Metabolite Analysis

Comprehensive profiling and targeted metabolite analysis of BAs both in rats’ serum and liver tissues were performed by Metabo-Profile Inc. (Shanghai, China) according to the previously published methods with minor modification [20]. The preparation of the serum sample was as follows: 50 µL of serum sample was added to a 1 mL 96-well plate, followed by 400 µL of acetonitrile/methanol (v/v = 8:2) mixed solution containing an internal standard. The 96-well plate was shaken at 650 rpm for 20 min at 10 °C. After centrifugation, 250 µL of the supernatant was transferred to 350 µL microplates and further lyophilized in a freeze dryer equipped with a stop tray system (Labconco, Kansas City, MO, USA). 40 µL acetonitrile/methanol (80/20) solution and 60 µL deionized water were added to the samples in turn, and shaken at 650 rpm for 20 min at 10 °C, respectively. After being frozen at –20 °C for 20 min, the samples were centrifuged at 4000 ×*g* for 30 min at 4 °C (Microfuge 20R, Beckman Coulter, Inc., Indianapolis, IN, USA) until measurements were taken.

The preparation of the liver tissue sample was as follows: 10 mg of liver tissue was added to an Eppendorf tube with a safety button; then, the tissue was mixed with 25 mg of precooled grinding beads and 20 µL ultrapure water by a homogenizer (BB24, Next Advance, Inc., Averill Park, NY, USA). 180 µL acetonitrile/methanol (v/v = 8:2) mixture containing 10 µL internal standard was added to tubes containing the sample, homogenized, and centrifuged at 13,500 rpm for 20 min at 4 °C (Microfuge 20R, Beckman Coulter, Inc., Indianapolis, IN, USA). The supernatant was then transferred to a 96-well plate and lyophilized in a freeze dryer equipped with a stop tray system (Labconco, Kansas City, MO, USA). The dried samples were redissolved with a 1:1 mixture of acetonitrile/methanol (80/20) and ultrapure water, and centrifuged at 13,500 rpm for 20 min at 4 °C (Microfuge 20R, Beckman Coulter, Inc., Indianapolis, IN, USA). The supernatant was then transferred to a 96-well plate for further analysis.

Quantitative analysis of BAs was performed with a UPLC-MS/MS, and the data were analyzed by MassLynx software (v4.1, Waters, Milford, MA, USA).

#### 2.2.8 Intestinal Flora Analysis by 16S RNA Sequencing

Intestinal flora comprehensive analysis and quantification of the fecal sample were performed by Majorbio Inc. (Shanghai, China) according to the previous methods with minor modifications. In brief, 1 g of fecal sample was added to 30 mL of sterile phosphate buffer saline (PBS), centrifuged at 1000 rpm for 5 minutes, and the process was repeated three times. The sediment was re-suspended in 10 mL PBS, and then the precipitate was collected. E.Z.N.A® Soil DNA kit (Omega Bio-Tek, Norcross, GA, USA) was used to extract the genomic DNA of microbial communities from the above samples according to the manufacturer’s instructions. Genomic DNA was detected by 1.0% agarose gel electrophoresis. The V3–V4 region of 16s ribosomal RNA gene was selected for amplification with a primer of 338F (ACTCCTACGGGAGGCAGCAG)/806R (GGACTACHVGGGTWTCTAAT) by an ABI GeneAmp® 9700 PCR thermocycler (ABI, CA, USA). PCR products were detected by 2% agarose gel electrophoresis [11]. The data were further analyzed using a free online tool on the Majorbio cloud platform (https://www.majorbio.com/).

## 3. Results

### 3.1 Potential Biomarkers and Metabolic Pathways in Rats’ Lipid Metabolites

**Fig. 2.**
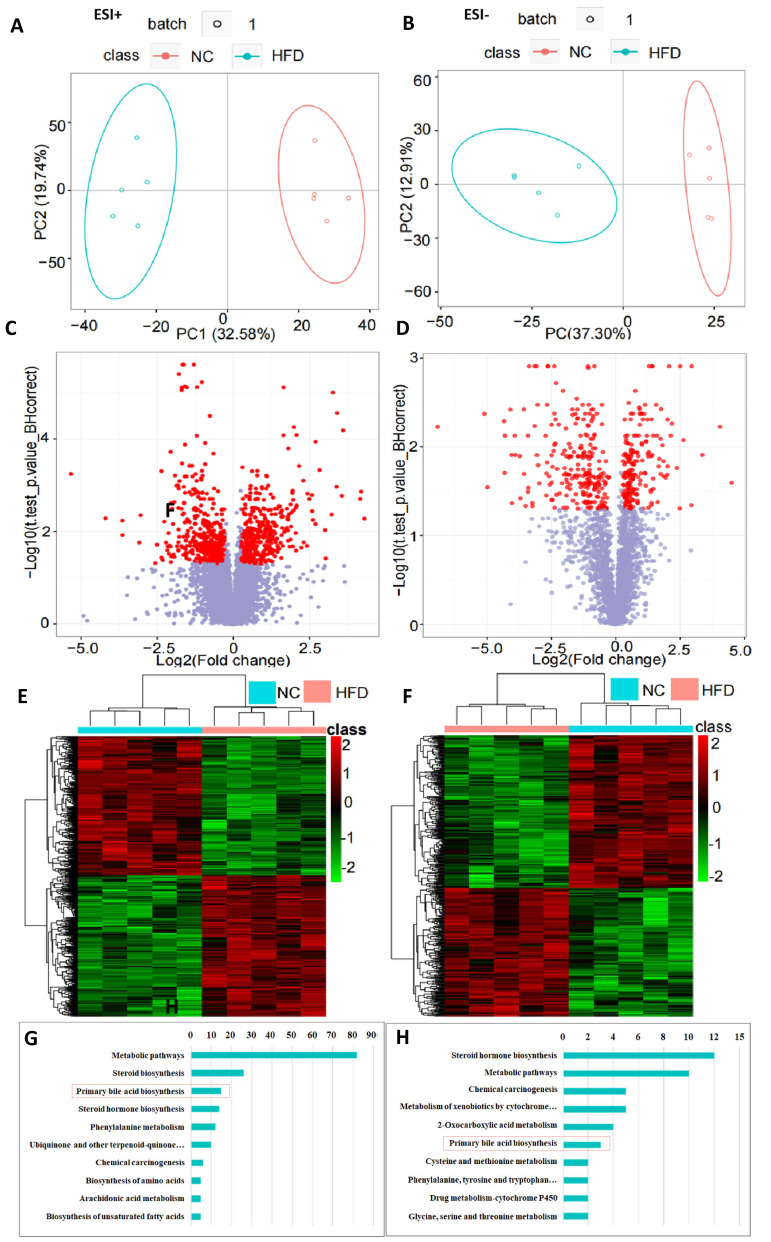
**Results of serum lipid metabolites and potential pathways influenced**. (A,B) PCA of metabolites in ESI+ and ESI–, respectively. (C,D) Volcano of metabolites in ESI+ and ESI–, respectively. (E,F) Heatmap of metabolites in ESI+ and ESI–, respectively. (G,H) Related metabolic pathways of differential metabolites in ESI+ and ESI–, respectively (RSD ≤30%, *p* < 0.05). Note: ESI+, electrospray ionization-positive ion mode; ESI–, electrospray ionization-negative ion mode; PC, principal component; PCA, principal component analysis; NC, negative control; HFD, high fat diet; RSD, relative standard deviation.

The blood lipid profiles were detected using UPLC-QTOF-MS. Numbers of differential metabolites in ESI+ and ESI– are shown in Table 1 and Fig. 2C,D. PCA and heatmap were applied to determine the metabolic distinction between the normal and HLP model groups. As shown by the score plots in Fig. 2A,B, the metabolic profiles of serum in ESI+ and ESI– from the normal and HFD model groups were clearly separated, whereby the samples in the HFD model group were significantly separated those in the normal group. These results indicate that there was a significant difference in blood metabolite profiles between the two groups.

**Table 1.** **Differential ions and identification**.

Mode	Diff number	Up (MS)	Down (MS)	Up (MS2)	Down (MS2)
ESI+	811	264	233	156	148
ESI–	456	57	110	24	51

Note: MS, mass spectrometry; ESI+, electrospray ionization-positive ion mode; ESI–, electrospray ionization-negative ion mode.

Among the ions in ESI+ and ESI–, with BH correct *p* values below 0.005, mass error lower than –5, and ratio of normal/model under 0.5, six metabolites were selected and identified as potential biomarkers, including a BA compound: tauroursocholic acid (Table 2). Based on the KEGG database (http://www.genome.jp/kegg/), we attempted to predict the potential signal pathway that has altered in hyperlipidemia. As shown in Fig. 2G,H, metabolic pathways, steroid synthesis, primary BA synthesis, and steroid hormone synthesis were the main pathways affected by HFD intake. Combined with the results of the differential metabolites analysis, we hypothesized that the BA metabolism pathway would be a potential intervention pathway for hyperlipidemia.

**Table 2.** **Potential biomarkers in blood associated with hyperlipidemia based on UPLC-QTOF-MS analysis**.

Mode	RT	m/z	Formula	Metabolite	N/M
ESI+	11.60	861.6312	C53H90O6	TG	0.475
	11.62	243.2118	C15H30S	2-N-Undecyltetrahydrothiophene	0.310
	11.63	231.2086	C17H28O	Heptadecatrienal	0.323
	11.63	509.3004	C12H26N8O3	Polyarginine	0.407
	11.66	720.6606	C49H82O2	Cholesteryl ester	0.285
ESI–	8.07	626.5226378	C38H73NO3	Cer (d16:2(4E,6E)/22:0)	0.474
	6.48	479.3621425	C30H52O2	Dammarenediol-I	0.119
	6.47	514.2801732	C26H45NO7S	Tauroursocholic acid	0.241
	6.47	653.2885352	C35H46N4O4S	BILA 2185BS	0.201
	9.46	697.513068	C40H75O7P	PA (P-20:0/17:2(9Z,12Z))	0.058
	9.57	780.5860696	C45H84NO7P	PE (O-20:0/20:4(5Z,8Z,11Z,14Z))	0.313

Note: UPLC-QTOF-MS, Ultra Performance Liquid Chromatography-Quadrupole-Time of Flight-Mass Spectrometry; RT, retention time; N/M, normal versus model; ESI+, electrospray ionization-positive ion mode; ESI–, electrospray ionization-negative ion mode; TG, triglycerides; Cer (d16:2(4E,6E)/22:0), N-(docosanoyl)-4E, 6E-hexadecasphingadienine; BILA 2185BS, (2S,4R)-N-(tert-Butyl)-1-((2R,3S)-3-(2-(2,6-dimethylphenoxy)acetamido)-2-hydroxy-4-phenylbutyl)-4-(pyridin-4-ylthio)piperidine-2-carboxamide; PA (P-20:0/17:2(9Z,12Z)), 1-(1Z-eicosenyl)-2-(9Z,12Z-heptadecadienoyl)-glycero-3-phosphate; PE (O-20:0/20:4(5Z,8Z,11Z,14Z)), 1-eicosyl-2-(5Z,8Z,11Z,14Z-eicosatetraenoyl)-glycero-3-phosphoethanolamine.

### 3.2 Specific Intestinal Flora Influencing BA Metabolism in Hyperlipidemia

Recent studies have shown that intestinal flora play a pivotal role in hyperlipidemia and BA metabolism [11, 21]. In the present study, 16S RNA was applied to screen the main types of intestinal flora that influence BA metabolism in hyperlipidemia.

**Fig. 3.**
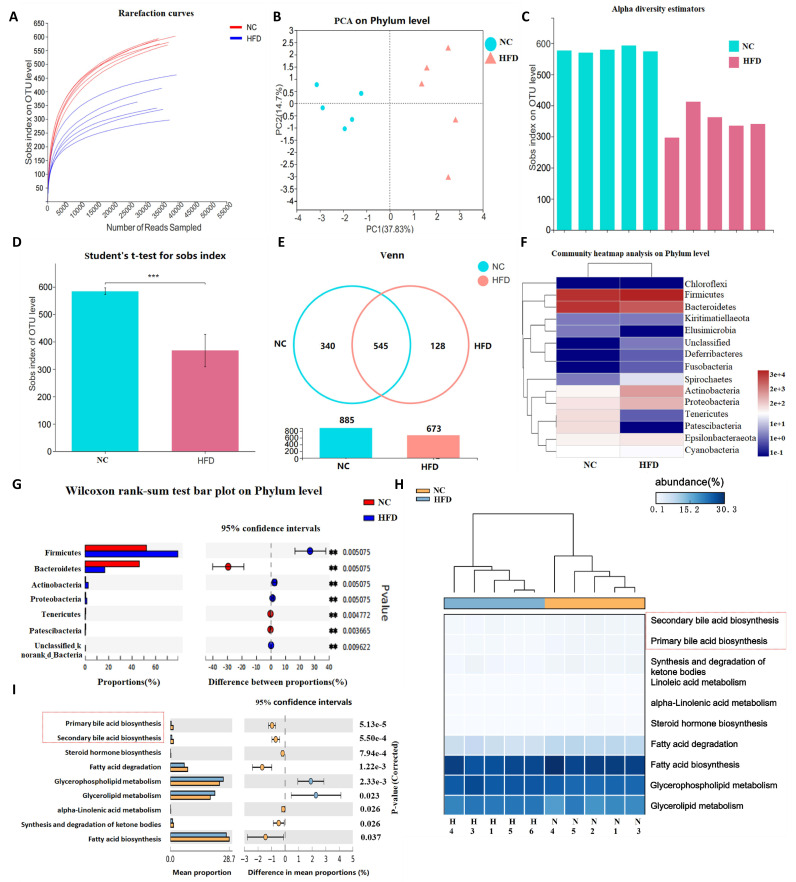
**Results of intestinal flora analysis**. (A) Dilution curve. (B) Principal component analysis (PCA). (C,D) OTU level and significance test. (E) Number of differential strains. (F) Heat map of differential strains. (G) Significance test of major differential strains. (H) Heat map of differential pathway. The pathways of secondary bile acid biosynthesis and primary bile acid biosynthesis were included. (I) Significance test of primary bile acid biosynthesis and secondary bile acid biosynthesis, the two major differential pathways. Note: OTU, operational taxonomic unit; NC, normal control; HFD, high fat diet.

The dilution curve of the number of reads sampled and the Sobs index at the Operational Taxonomic Units (OTU) level showed that the sampling depth is sufficient for differential analysis (Fig. 3A). PCA analysis demonstrated that samples in the HFD group were significantly separated from those in the normal group, indicating a significant difference between the two groups at the gate level (Fig. 3B). The difference was verified by the Sobs index of OTU levels (Fig. 3C,D). The VENN figure showed 545 shared microflora between the two groups, 340 in the NC group, and 128 in the HFD group (Fig. 3E). The heatmap of the different flora revealed that, compared with the normal group, the abundance of Bacteroidetes, Elusimicrobia, Tenericutes, and Patescibacteria was decreased, while Fusobacteria, Spirochetes, Actinobacteria, and Proteobacteria was significantly increased in the HFD group (Fig. 3F). The Wilcoxon rank-sum test confirmed the significance at the phylum level (Fig. 3G). Functional annotation results showed that the functions of the above differential flora were mainly related to primary and secondary BA metabolism (Fig. 3H,I).

### 3.3 BA Metabolism Analysis

Analysis of both serum lipid metabolism and the intestinal flora suggest that the observed results were indicative of the process of BA metabolism. To verify the change in the BA metabolite profile in HFD-induced HLP, and to determine whether there was a specific BA that was closely related to HLP and can be used as a potential therapy target or candidate drug for HLP, we conducted metabolomic analyses of BA both in serum and liver tissue of normal rats and HLP rats.

**Fig. 4.**
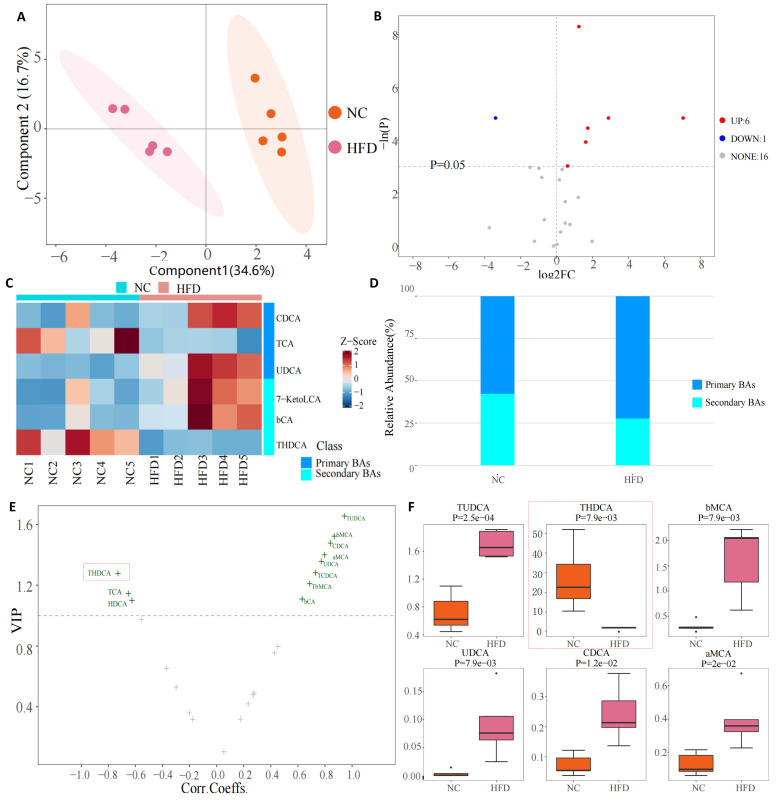
**Bile acid metabolites in liver tissue**. (A) PCA of two groups. (B) Volcano of different metabolites between two groups. (C) Bile acids heatmap. (D) Relative abundance of primary and secondly bile acid. (E) VIP plot of bile acid changes. (F) Boxplot of specific bile acid. Note: NC, negative control; HFD, High fat diet; THDCA, taurohyodeoxycholic acid; TCA, taurocholic acid; bCA, 3β-cholic acid; BAs, bile acids; TUDCA, tauroursodeoxycholic acid; bMCA, Tauro-β-muricholic acid; UDCA, ursodeoxycholic acid; CDCA, chenodeoxycholic acid; aMCA, a-Muricholic Acid; PCA, principal component analysis; VIP, variable importance in the projection; FC, fold change.

Metabolite analysis of BAs in the liver tissue of rats is shown in Fig. 4. PCA analysis showed a significant difference between the NC and HFD groups (Fig. 4A). An increased abundance of six metabolites and decreased levels of one metabolite were found in the volcano of different metabolites (Fig. 4B). A Heatmap was drawn to obtain the results of the metabolites of BAs (Fig. 4C). The results showed an increase in the levels of α-muricholic acid (αMCA), βMCA, chenodeoxycholic acid (CDCA), Tauro-β-muricholic acid (TbMCA), tauroursodeoxycholic acid (TUDCA), and ursodeoxycholic acid (UDCA), and a decrease in THDCA. Compared with the NC group, the levels of secondary BA in the HFD group significantly decreased (Fig. 4D). In secondary BAs, THDCA was the metabolite that was significantly decreased (Fig. 4E,F).

**Fig. 5.**
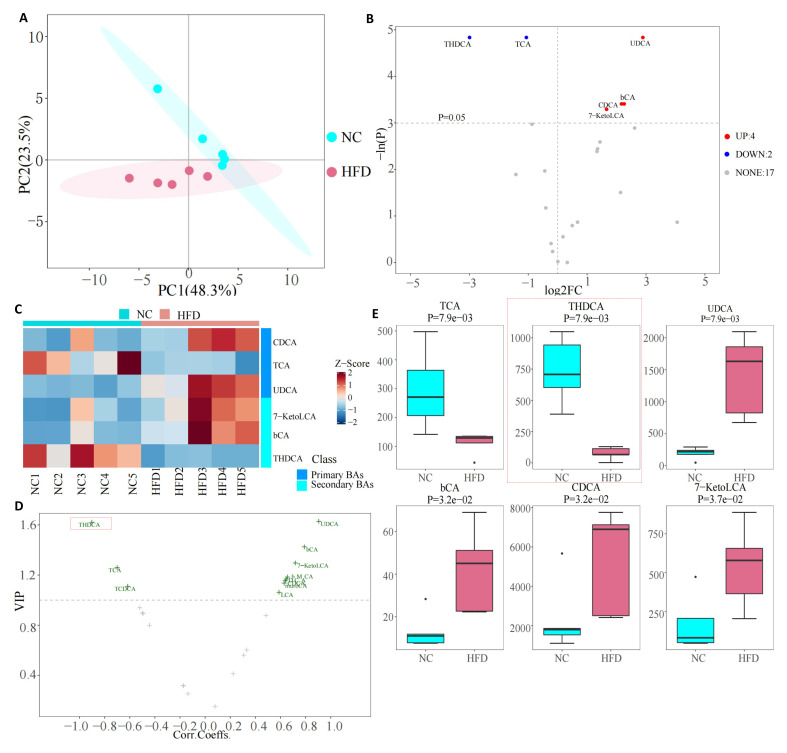
**Bile acid metabolites in serum**. (A) PCA of bile acids in the NC and the HFD group. (B) Volcano of bile acids of the NC and HFD groups. (C) Heatmap of bile acids of the NC and HFD groups. (D) VIP plot of bile acids changes. (E) Boxplot of specific bile acid. The level of THDCA in serum of rats in the HFD group is lower than that in the NC group. Note: NC, normal control; HFD, high fat diet; PC, principal component; THDCA, taurohyodeoxycholic acid; TCA, taurocholic acid; UDCA, ursodeoxycholic acid; bCA, 3β-cholic acid; CDCA, chenodeoxycholic acid; 7-KetoLCA, 7-ketolithocholic acid; PCA, principal component analysis; VIP, variable importance in the projection; FC, fold change.

The results of the analysis of serum BA metabolites are shown in Fig. 5. PCA analysis also revealed a significant difference in serum BA metabolites between the NC and HFD groups (Fig. 5A). An increased abundance of 4 metabolites and decreased levels of 2 metabolites were found in the volcano of different metabolites (Fig. 5B). Heatmap drawings showed that the differential metabolites of the HFD group compared with the NC group were related to both primary and secondary BAs (Fig. 5C), among which expressions of CDCA, UDCA,7-Ketolithocholic Acid (7-KetoLCA), and 3β-Cholic Acid (bCA) increased, while TCA and THDCA levels decreased. Comparative analysis of the specific BAs also found the same results, namely, that THDCA in the secondary BAs decreased significantly (Fig. 5D,E).

### 3.4 Verification Experiments Confirmed the Function of THDCA on HLP Treatment

Based on the results obtained from the lipid profiles, intestinal flora analysis, and BA metabolite detection, a hypothesis was formed that the development of hyperlipidemia was primarily attributed to the impact of a high-fat diet on THDCA, a secondary BA. To validate the beneficial effect of THDCA on anti-HLP, we established an HLP mouse model and an intestinal dysbacteriosis mouse model (IFI) to verify the functions of THDCA on blood lipid lowering and intestinal flora disequilibrium.

**Fig. 6.**
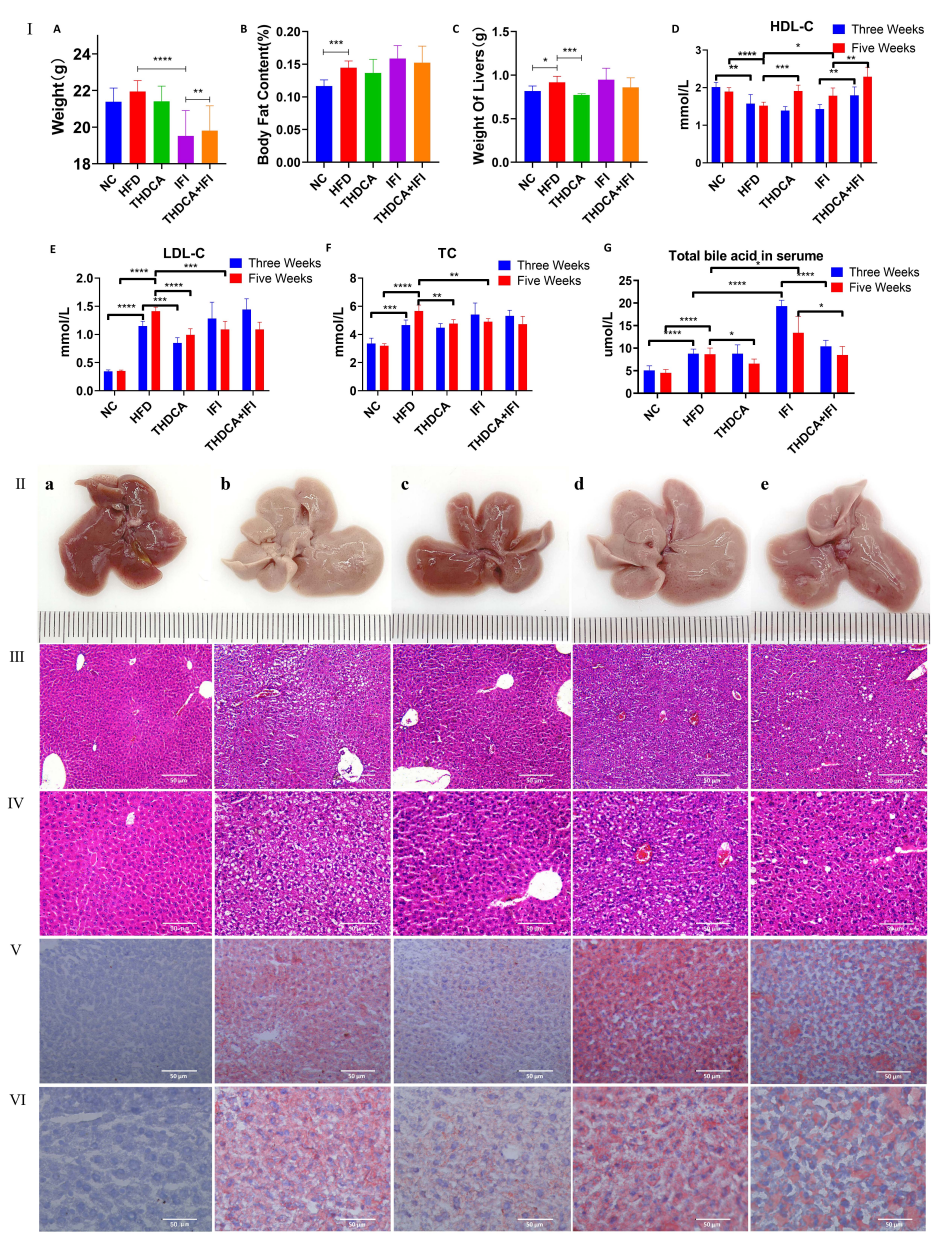
**Effects of THDCA on anti-hyperlipidemia**. (I) Effect of THDCA on body weight and levels of serum lipids: (A) Final body weight. (B) Body fat of mice. (C) Liver weight of mice. (D) Serum HDL-C levels at 3 and 5 weeks. (E) Serum LDL-C levels at 3 and 5 weeks. (F) Serum TC level changes at 3 and 5 weeks of administration. (G) Serum total bile acids at 3 and 5 weeks. (*p < 0.05, **p < 0.01, ***p < 0.001, ****p < 0.0001). (II) The overall morphology of the livers; H&E staining of liver tissue (III: 100×, IV: 200×), and oil red O staining of liver tissues (V: 200×, VI: 400×). (a) normal group. (b) HFD group. (c)THDCA 100 mg/mL group. (d) microflora dysregulation group. (e) THDCA + microflora dysregulation group. The bar is 50 µm (mean ± SD). Note: HDL-C, high-density lipoprotein cholesterol; LDL-C, low-density lipoprotein cholesterol; TC, total cholesterol; NC, normal control; HFD, high fat diet; THDCA, taurohyodeoxycholic acid; IFI, intestinal flora imbalance.

The results of the verification experiments are shown in Fig. 6. It was found that the body fat of mice in the HFD group was significantly higher than that of the NC group (Fig. 6I,B). Compared with the NC group, serum TC and LDL-C levels of mice in the HFD group increased significantly, while HDL-C levels decreased significantly (Fig. 6I,D–F). Abnormal TBA levels were also found in mice in the HFD and IFI groups (Fig. 6I,G). H&E staining and red oil O staining both confirmed the characteristics of hyperlipidemia, such as liver swelling, enlarged hepatocytes, and lipid deposition in hepatocytes in mice induced by HFD and antimicrobial agents (Fig. 6II–VI).

Comparison of the HFD and THDCA groups was carried out during the same period (Fig. 6I,A–C). Liver weight of mice in the HFD group was significantly higher than that in THDCA group. Compared with the HFD group, higher HDL-C levels and lower TC, TBA levels at weeks five, and lower LDL-C were found in the THDCA group. H&E staining (Fig. 6III,IV) and oil red O staining (Fig. 6V,VI) of liver tissue confirmed that THDCA supplementation could effectively retard the development of hyperlipidemia and reduce lipid deposition in liver tissue (Fig. 6I,D–F,II,III).

In the comparison between the IFI group and HFD group (Fig. 6I,A–G) over the same period, an increased weight was found in IFI mice. The IFI group exhibited higher serum levels of TBA and HDL-C at week five, along with lower levels of LDL-C and TC at week five. These findings suggest that dysbiosis may play a role in the development of hyperlipidemia. THDCA intervention greatly improved the abnormal levels of blood lipids augmented by dysbiosis, and blocked the development of HLP.

## 4. Discussion

Dietary intake has a significant impact on human metabolism. A wealth of evidence suggests that an excessive intake of wine, carbohydrates, and fats commonly contained in the diet, is linked to the development of hyperlipidemia [22]. Currently, the primary pharmaceutical interventions for hyperlipidemia include statins, niacin, and fibrates. Statins, in particular, are the most widely prescribed medication for patients with hyperlipidemia. Statins reduce plasma LDL-C uptake by inhibiting HMG-CoA reductase. However, statins carry side effects, such as myositis, resulting in more severe kidney injury [8]. Niacin was found to regulate lipids in 1962, but has not been widely used due to side effects such as skin flushing, rash, gastrointestinal discomfort, hyperuricemia, hyperglycemia, and liver dysfunction [8]. Fibrates cause gastrointestinal reactions, headaches and insomnia. Thus, investigating the mechanism of diet-induced hyperlipidemia and finding new clinical drugs is crucial. Research shows that gut flora plays a key role in metabolism regulation and has a significant impact on various chronic diseases [23]. The use of high-throughput technology in the study of gut flora in hyperlipidemia shows that changes and disorders in the gut environment and flora result from abnormal lipid metabolism. Recent studies have analyzed the human intestinal flora, and found that the flora has a significant impact on lipid levels and BMI [24]. Microflora can, in turn, regulate the homeostasis of lipid metabolism and promote the development of hyperlipidemia.

In our study, we found that HFD can lead to the increase of Firmicutes and the decrease of Bacteroidetes. Imbalance of the F/B ratio has been recognized as a key factor in the development of hyperlipidemia. Our results also showed that after an HFD diet, there is a significant rise in F/B, which is consistent with previous studies [25, 26]. Moreover, our results demonstrate that Actinobacteria are also elevated after hyperlipemia. The gut’s rare Actinobacteria species have been associated with disorders of nonalcoholic fatty liver lipid metabolism [27]. Cholesterol absorption, synthesis, catabolism, and lipoprotein transport in the liver are important factors in maintaining the homeostasis of lipid metabolism. BAs, synthesized from cholesterol, constitute the main catabolic route of cholesterol in the human body [28]. They also serves as a potent regulator of lipid and cholesterol metabolism [29]. HDL transports cholesterol from tissues to the liver for BA synthesis and excretion. This process facilitates lipid digestion [30]. The regulation of lipid metabolism by BAs is achieved through the activation of TGR5 and FXR receptors [31].

BAs have been shown to increase energy consumption in adipose tissue and muscle in mice by activating TGR5 in brown adipocytes and its downstream signaling pathways. This highlights the crucial role that BAs play in regulating lipid metabolism in the body. Intestinal flora can also regulate lipid metabolism by balancing the bile acid pool and composition. In the terminal ileum and colon, primary BAs are converted into secondary BAs by the microbially associated bile salt hydrolase and 7-dehydrogenase [32]. BA synthesis is then negatively regulated by the activation of FXR in the ileum and liver. Therefore, in mice, the gallbladder of the sterile group was enlarged, and more primary BAs were secreted [33].

According to our analysis, HFD caused abnormal metabolism of primary and secondary BAs. Further analysis showed that UDCA and CDCA in primary BAs were elevated in both serum and liver. UDCA and CDCA are well-known BA types that have been developed for clinical use. UDCA is considered the gold standard treatment for primary biliary cirrhosis, but its efficacy in the treatment of hyperlipidemia has not been validated [34, 35]. CDCA has been shown to have a potential therapeutic effect on hyperlipidemia, as it inhibits cholesterol absorption [36]. However, after a HFD diet, the serum and liver levels of CDCA have been adaptively increased, so the benefit of additional supplementation of CDCA will be reduced. It has been observed that THDCA is the only secondary BA that exhibits a decrease both in serum and liver in HLP animals. THDCA is a hydrophilic BA that can stimulate the secretion of cholesterol and phospholipids into bile [37]. Studies have revealed that THDCA has the ability to enhance the expression of genes related to the apolipoprotein family and lipid metabolism, reduce lipid accumulation, and enhance the insulin sensitivity of hepatocytes in a dose-dependent manner [38]. In addition, THDCA is an agonist of FXR, which can reverse hepatic steatopathy and prevent liver injury by stimulating FXR and its downstream metabolism.

The results of our study indicate that THDCA possesses a therapeutic effect for the treatment of hyperlipidemia by controlling lipid metabolism via the modulation of BA metabolism. THDCA can effectively reduce body weight, lower body fat percentage, LDL-C, and TC in the blood, and also have a therapeutic effect on dyslipidemia caused by bacterial imbalance. THDCA may hold potential as a direct therapeutic agent for the treatment of hyperlipidemia, or it may be combined with statins to enhance their efficacy in the treatment of hyperlipidemia. This study identified THDCA as a central player in the formation of dietary hyperlipidemia through a multi-omics examination, and uncovered the potential relationships between THDCA, lipid metabolism, intestinal microflora, and BA metabolism. Further in-depth experiments and clinical studies are needed to prove its effectiveness, and to determine its mechanism.

## 5. Conclusions

By mimicking the high-fat diet in humans, we established an HLP animal model. Through comprehensive profiling of serum and liver BAs, as well as gut microbiota, we found that reduction of THDCA is critical for the development of hyperlipidemia in response to a high-fat diet. We validated this result by animal experiments in mice and suggested THDCA should be investigated as a potential therapeutic agent for hyperlipidemia.

## Data Availability

The datasets used and/or analyzed during the current study are available from the corresponding author on reasonable request.
